# Research progress on *Alpinia oxyphylla* in the treatment of diabetic nephropathy

**DOI:** 10.3389/fphar.2024.1390672

**Published:** 2024-06-14

**Authors:** Jing Wang, Xiaomin Wang, Tianpeng Ma, Yiqiang Xie

**Affiliations:** ^1^ Hospital of Chengdu University of Traditional Chinese Medicine, Chengdu, China; ^2^ Hainan Medical University, Haikou, Hainan, China

**Keywords:** diabetic nephropathy, *Alpinia oxyphylla*, Chinese herbal medicine, mechanism, research progress

## Abstract

Diabetic nephropathy (DN) constitutes a major microvascular complication of diabetes and is a primary cause of mortality in diabetic individuals. With the global rise in diabetes, DN has become an urgent health issue. Currently, there is no definitive cure for DN. *Alpinia oxyphylla*, a Chinese herbal medicine traditionally used, exhibits a wide range of pharmacological effects and is frequently used in the prevention and management of DN. This paper offers an extensive review of the biological mechanisms by which *A. oxyphylla* delivers therapeutic advantages in DN management. These mechanisms include activating podocyte autophagy, regulating non-coding RNA, modulating gut microbiota, alleviating lipotoxicity, counteracting oxidative stress, and diminishing inflammatory responses, underscoring the therapeutic potential of *A. oxyphylla* in DN treatment.

## 1 Introduction

Diabetic nephropathy (DN) constitutes a significant microvascular complication among diabetes patients, with 30%–40% estimated to develop DN over their lifetimes (Deshpande, Harris-Hayes, and Schootman, 2008). It is the leading cause of end-stage renal disease (Gupta, Dominguez, and Golestaneh, 2023), thus generating a considerable global economic impact. DN is clinically characterized by progressive proteinuria and a reduction in glomerular filtration rate (GFR) (Pelle et al., 2022). Present treatments primarily target modulating risk factors, such as blood pressure and glucose levels, and mitigating proteinuria. However, these therapies, when applied long-term, can lead to various side effects, including persistent dry cough and angioedema (Shen et al., 2017), and frequently prove ineffective in significantly halting DN progression. This underscores an urgent need to explore and validate effective treatments. Recent studies highlight the promising role of Chinese herbal medicine in DN management due to its unique advantages (Yang et al., 2020; Liu et al., 2022; Wang et al., 2023).

*Alpinia oxyphylla*, commonly known as Yizhi, has been esteemed for its medicinal properties since its initial mention in the “Kaibao Bencao.” In 1998, the Chinese Ministry of Health designated it as a plant with dual purposes: medicinal and edible (Kai et al., 2021a). Current studies reveal that *A. oxyphylla* primarily contains diarylheptanoids, flavonoids, sesquiterpenes, and glycosides (Wu et al., 2019). Pharmacological research has identified several properties of this plant, such as anti-inflammatory, anti-aging, antioxidant, antidiuretic, immunostimulatory, and neuroprotective activities (Wang et al., 2018; Yu et al., 2020; Zhang et al., 2020; Park et al., 2022; Wang et al., 2023). Its renal protective effects have gained considerable attention, with studies highlighting the efficacy of *A. oxyphylla* extract in combating DN. Notably, the petroleum ether extract appears to be the most effective (Xie et al., 2015). Early intervention with *A. oxyphylla* and its extracts can decelerate DN progression, as shown by the alleviation of polyuria, reduction in 24-h urine protein levels, blood urea nitrogen (BUN), serum creatinine (Scr), and improvement in glomerular hypertrophy, mesangial expansion, and basement membrane thickening in DN mice (Xie et al., 2015; Chen et al., 2016). The critical aspect is that Suoquan Yishen Fang, primarily consisting of *A. oxyphylla*, is a traditional Chinese medicine compound that has been granted a national patent (patent number: ZL 2016 1 0423677.5) for treating DN. Studies have indicated that the Suoquan Yishen Fang can mitigate the clinical symptoms of DN and decrease urea nitrogen and urine protein levels in patients (Yin et al., 2019). This article examines the molecular mechanism of action of *A. oxyphylla* in DN, exploring potential strategies for mitigating and reversing renal injury, thereby informing future research and product development efforts in the treatment of DN with *A. oxyphylla*.

## 2 Roles of *Alpinia oxyphylla* on DN

**FIGURE 1:**
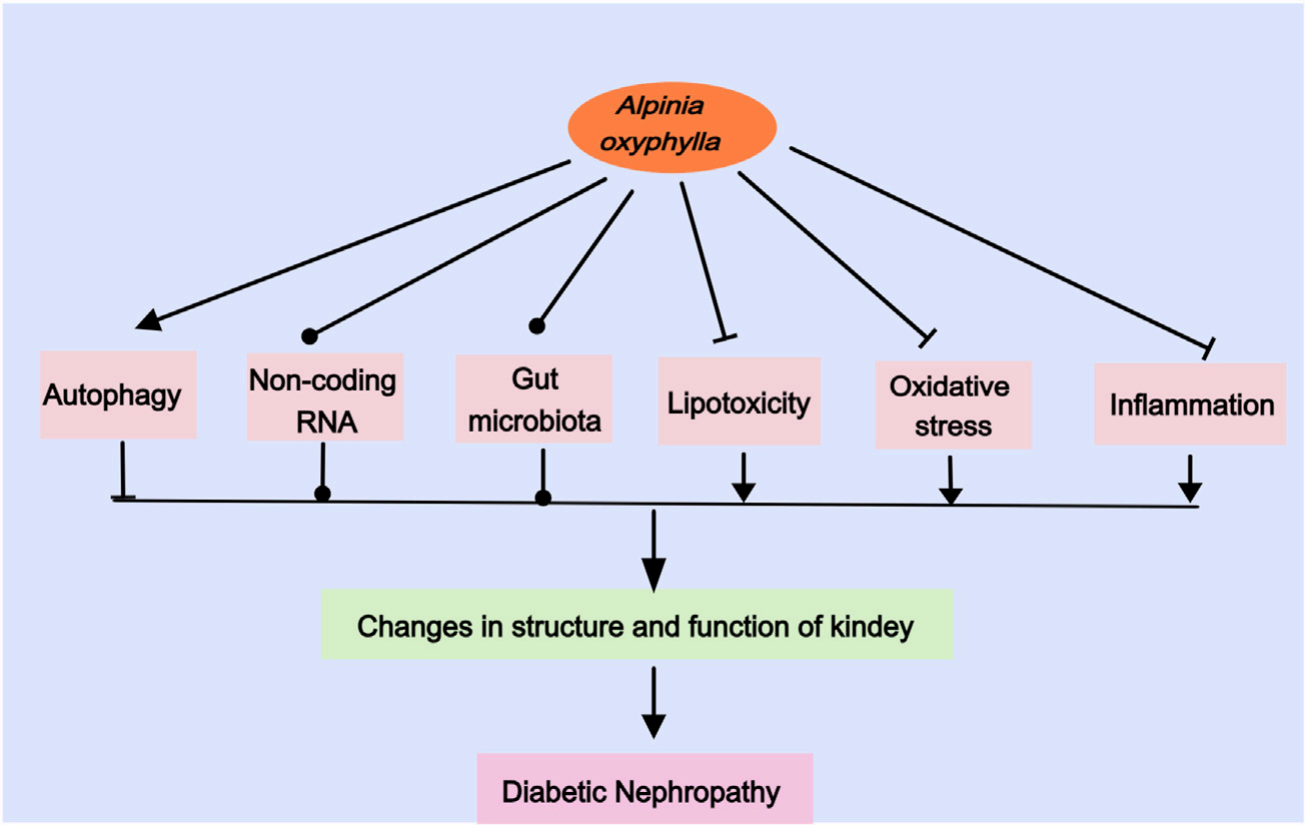
Roles of *Alpinia oxyphylla* on DN.

A plethora of evidence indicates that mechanisms such as autophagy, non-coding RNA, the gut microbiome, lipotoxicity, oxidative stress, and inflammatory responses contribute to DN (Cui et al., 2020; Opazo-Ríos et al., 2020; Jin et al., 2023; Szostak et al., 2023; Zhao et al., 2023). Recent pharmacological studies have revealed that *A. oxyphylla* possesses therapeutic capabilities, including the activation of autophagy, regulation of non-coding RNA, modulation of intestinal microbiota, attenuation of lipotoxicity, reduction of oxidative damage, and mitigation of inflammatory responses (Du et al., 2017; Xie et al., 2017; Xie et al., 2018; Wang et al., 2022; Wang et al., 2023c; Ni et al., 2023; Yin et al., 2023). Such findings underscore *A. oxyphylla,* whether as a single agent, an extract, or in combination, as a promising multipronged intervention for DN, targeting various underlying mechanisms (Figure 1) (Table 1).

**TABLE 1:** The role of *Alpinia oxyphylla* (single drug, extract and combination) on DN.

Type of action	Results	Experiment type	Model	References
Activating podocyte autophagy	GBM↓ podocyte autophagosomes↑	Animal testing	DN mice	Yin et al. (2023)
	LC3↑ Atg5↑Becn1↓P62↓PI3K↓ Akt↓ mTOR↓			
	Autophagy ↑	Animal testing cellular assays	DN mice	Yin et al. (2021)
			Podocyte	
Regulation non-coding RNA	miRNA-21↓p-AKT↓AKT↓PI3K↓ PTEN↑	Animal testing cellular assays	DN mice, GMCs	Mao et al. (2018)
	let-7k、miR-378d、miR-129-1-3p、miR-21a-5p、miR-29c-3p、miR-203-3p and miR-7a-5p↑ Glu↓ Scr↓	Animal testing	DN mice	Du et al. (2017)
	UAER↓ UACR↓			
	miR-598↑miR-423↑PTEN↓TG↓TC↓ Glu↑ INS↑ Renal function↑	Animal testing	DN mice	Yang (2018)
	LncRNA-PVT1↓LN↓FN↓COI-IV↓ MMP-9↑TIMP-1↓TGF-β1↓	Animal testing	DN mice	Jiaxin, (2021)
	GM ↓ GS ↓ KDM6A↓ KLF10↓	Animal testing cellular assays	DN mice	Wang, (2023)
	α-SMA↓ N-cadherin↓ P-cadherin↑ nephrin ↑		Podocyte	
Modulating gut microbiota	IMD↑*Firmicutes*↓	Animal testing	DN mice	Ni et al. (2021)
	*Bacteroidetes*↑ *Akkermansia* ↑			
	*Proteobacteria*↓Enterobacteriaceae↓ Prevotellaceae↑ *Romboutsia* ↑	Animal testing	DN rats	Wu, (2021)
	Verrucomicrobiaceae*↑* Lactobacillaceae*↑* Rikenellaceae *↓*Lachnospiraceae*↓ Akkermansia↑ Bacteroides↑Helicobacter↓ Blautia↓*	Animal testing	DN mice	Xie et al. (2018)
	*Cyanobacteria*↓ *Deferribacteres*↓	Animal testing	DN mice	Ni (2019)
	*Verrucomicrobia*↓ Porphyromonadaceae↑			
	*Intestinimonas*↑ *Mucispirillum*↑			
	*Parabacteroides*↑ *Alistipes*↑			
	*Akkermansia*↑ Prevotellaceae↑			
	Lachnospiraceae↑ *Anaerotruncus*↑			
	Ruminococcaceae↑ *Bacteroides*↑			
Ameliorating lipotoxicity	SM↓PC ↓LPC↓PE↓	Animal testing	DN mice	Ni et al. (2023)
Combating oxidative stress	MDA↓ GSH↑ POD↑SOD↑UAE↓	Animal testing	db/db mice	Xie et al. (2017)
	UCr ↓BUN↓TG↓TC↓			
	SOD↑CAT↑GSH↑ MDA↓	Animal testing	DN mice	Mo et al. (2016)
Mitigating inflammatory responses	NLRP3 ↓caspase-1↓ IL-18 ↓IL-1β↓	Animal testing cellular assays	DN mice	Kai et al. (2021b)
			HK-2	
	HK-2 survival rate↑ NLRP3↓	Animal testing cellular assays	DN mice HK-2	Wang et al. (2022)
	IL -18 ↓IL - 1β↓			

### 2.1 Activating podocyte autophagy

Autophagy, a self-degradative process in eukaryotic cells, involves lysosomal elimination of cytoplasmic proteins and impaired organelles, regulated by autophagy-related genes (Atg) (Lahiri et al., 2019). This mechanism operates continuously under normal physiological conditions—known as basal autophagy—and intensifies in response to cellular stress (Galluzzi and Green, 2019). As a protective cellular mechanism, autophagy contributes to cell growth and resilience, shielding cells from metabolic strain and oxidative harm, thereby playing a pivotal role in sustaining intracellular equilibrium as well as the synthesis, degradation, and recycling of cellular constituents (Lahiri et al., 2019). However, excessive activation of autophagy may lead to metabolic imbalances, unnecessary degradation of cellular structures, and potentially result in cell death (Xu and Hu, 2022).

Research has demonstrated that autophagy plays a crucial role in DN by maintaining glomerular and tubular homeostasis (Liu et al., 2022; Liu et al., 2022; Su et al., 2022). Unlike other glomerular cells, podocytes cannot remove harmful substances through cellular division due to their terminally differentiated state, necessitating increased basal autophagy for their elimination (Sun et al., 2021). As a result, these cells exhibit enhanced basal autophagic activity. However, chronic exposure to high glucose conditions leads to a reduced autophagic response in podocytes, significantly compromising their protective mechanism and aggravating cellular injury (Xu et al., 2021). Notably, such damage to podocytes is a critical factor in the progression of proteinuria and DN (Barutta et al., 2022; Li et al., 2023).

**FIGURE 2:**
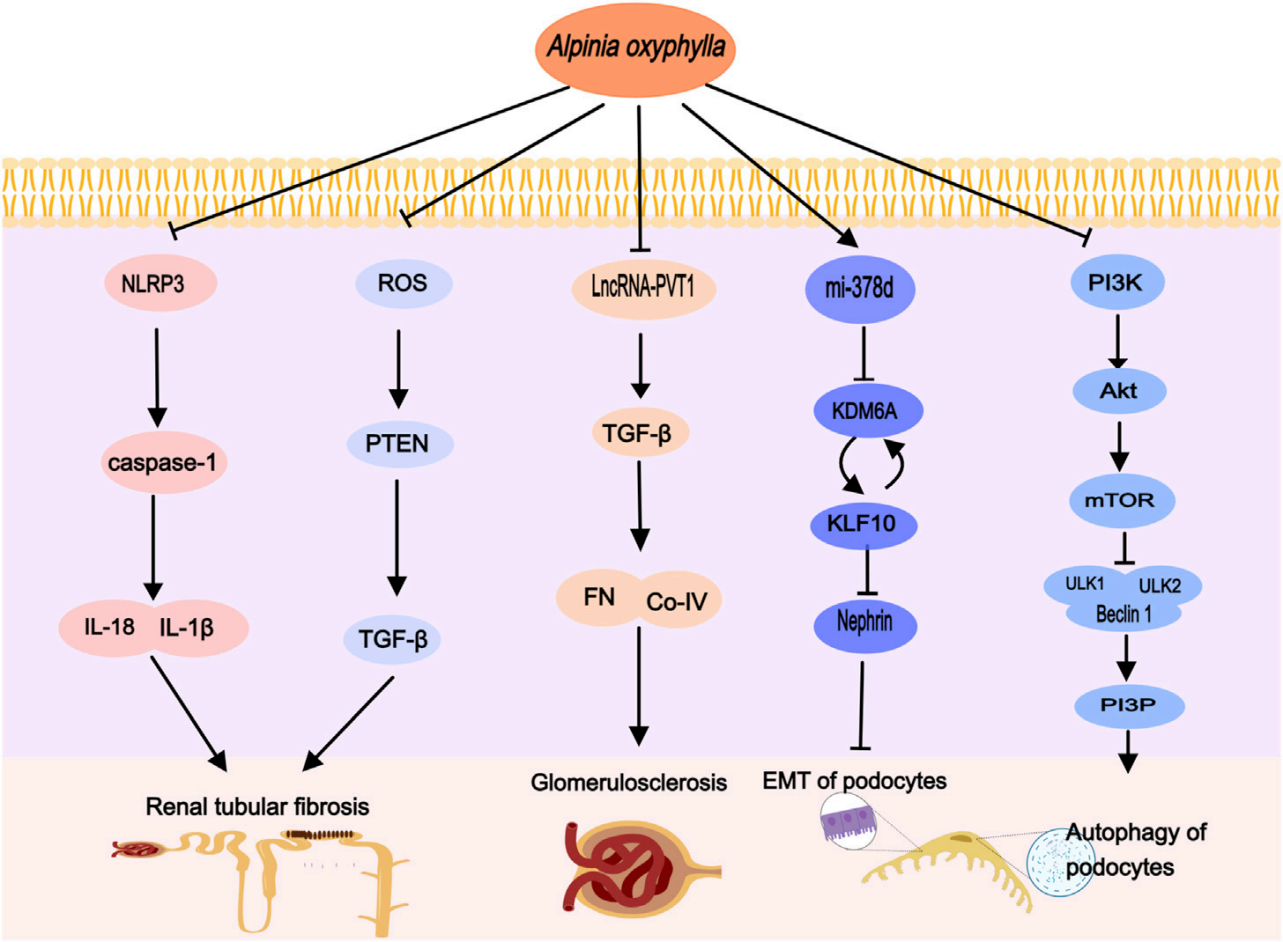
*Alpinia oxyphylla* treats DN by regulating the above signaling pathways, activating podocyte autophagy, inhibiting EMT, improving glomerulosclerosis and renal tubular fibrosis.

Recent research indicates that the phosphatidylinositol-3-kinase/protein kinase B/mammalian target of rapamycin (PI3K/Akt/mTOR) signaling pathway plays a crucial role in podocytes, primarily by downregulating autophagy (Yang X et al., 2020; Lai et al., 2023). Following DN, elevated levels of inflammatory factors and reactive oxygen species (ROS) can activate cell surface receptors, initiating the PI3K activation process. This activation of PI3K leads to downstream activation of Akt and mTOR (Miricescu et al., 2021). Moreover, mTOR phosphorylates several autophagy-related proteins, such as UNC-51 like kinase 1 (ULK1), inhibiting critical autophagy complexes and thereby impeding autophagosome formation and affecting podocyte autophagy (Al-Bari and Xu, 2020; Yang et al., 2023). A study by Yin Dehui et al. on DN mice revealed that *A. oxyphylla* suppresses the activation of the PI3K/Akt/mTOR pathway, enhances podocyte autophagy, mitigates podocyte damage (Yin et al., 2023), and may provide therapeutic benefits for DN (Figure 2).

### 2.2 Regulating non-coding RNA

Noncoding RNAs (ncRNAs) constitute a broad category of RNAs that do not encode proteins (Liu et al., 2021). This group includes diverse types of ncRNAs, with microRNA (miRNA), long noncoding RNA (lncRNA), and circular RNA (circRNA) as the primary functional classes. These have been shown to influence the pathogenesis of DN by regulating gene expression (Lv et al., 2020). This review will focus on the regulatory roles of miRNAs and lncRNAs in DN and investigate the therapeutic potential of *A. oxyphylla* in modulating these ncRNAs to treat DN.

#### 2.2.1 MicroRNA

MiRNAs, encompassing 19–22 base pairs, are single-stranded and function by downregulating gene expression through the targeted degradation of specific messenger RNAs (mRNAs) (Mauceri, 2022). Studies have demonstrated variations in miRNA expression levels when comparing kidney tissue samples from healthy individuals to those from patients with DN (Li et al., 2021). Similarly, research indicates differences in miRNA profiles within the kidneys of normal, *db/db* mice, and DN mice (Du et al., 2017; Yang, 2018; Zhu. et al., 2019). Notably, miRNA-378, which is under-expressed in the kidneys of *db/db* and DN mice compared to normal mice, is closely linked with insulin resistance and has the potential to modulate inflammatory stress alongside insulin resistance (Wang et al., 2021). Consequentl, miRNA-378 represents a promising target for the treatment of metabolic disorders (Machado et al., 2020).

It has been documented that *A. oxyphylla* is capable of restoring miR-378d expression in the kidneys of DN mice (Du et al., 2017). Concurrently, Suoquan Yishen Fang, which primarily contains *A. oxyphylla*, has been demonstrated to attenuate DN damage by upregulating miR-378d expression in the kidneys of *db/db* mice. This upregulation inhibits the histone demethylating enzyme KDM6A and the transcription factor KLF10, disrupting the epithelial-mesenchymal transition of podocytes (Wang, 2023). Additionally, *A. oxyphylla* has been recognized for enhancing the expression of six other microRNAs: let-7k, miR-129-1-3p, miR-21a-5p, miR-29c-3p, miR-203-3p, and miR-7a-5p (Du et al., 2017). Importantly, miR-21a-5p, miR-29c-3p, and miR-203-3p are implicated in podocyte damage and renal interstitial fibrosis, functioning through diverse signaling pathways (Long et al., 2011; Chen et al., 2018; Ji et al., 2019). This evidence suggests that *A. oxyphylla* may exert therapeutic effects on DN through the modulation of miRNA expression.

#### 2.2.2 Long noncoding RNA

lncRNAs, defined as ncRNAs longer than 200 nucleotides, play a pivotal role in gene regulation through interactions with proteins, DNA, and RNA (Yao et al., 2019; Ruffo et al., 2023). Extensive research has highlighted the significance of lncRNAs in the pathophysiological mechanisms underlying DN, involving glomerular and renal tubular injury, microvascular lesions, endoplasmic reticulum stress, inflammation, autophagy, apoptosis, and cell proliferation (Yi et al., 2017; Ebadi et al., 2019; Leung et al., 2019). Certain lncRNAs have been identified as prospective diagnostic markers and therapeutic targets for DN (Xu et al., 2022). Notably, Plasmacytoma Variant Translocation 1 (PVT1) was the first lncRNA associated with kidney disease and is characterized by increased expression in various kidney cell types (Hanson et al., 2007). Elevated PVT1 levels can enhance cellular proliferation and extracellular matrix (ECM) accrual, consequently accelerating DN progression (Qin and Cao, 2021). Glomerular mesangial cells (GMCs) are primary ECM secretors, proliferating and secreting components such as collagen type IV (Col-IV), fibronectin (FN), and laminin in response to high glucose, lipids, and filtration pressure (Alvarez et al., 2016). Transforming growth factor-β1 (TGF-β1) is recognized as a significant fibrogenic factor and a central pathway in renal fibrosis, critically involved in ECM buildup, renal fibrosis exacerbation, and DN progression (Lovisa et al., 2015). Elevated PVT1 expression in human mesangial cells (HMCs) under high glucose conditions, along with significant increases in FN1, COL4α1, TGF-β1, and plasminogen activator inhibitor-1 (PAI-1) levels, has been documented (Mok et al., 2022). Silencing PVT1 partially reverses high glucose-induced mesangial cell proliferation and fibrosis (Zhong et al., 2020), suggesting PVT1 as a promising target for DN treatment. Importantly, Suoquan Yishen Fang, primarily comprised of *A. oxyphylla*, reduces lncRNA-PVT1 and suppresses TGF-β1 and ECM components FN and Col-IV protein expression, thereby mitigating glomerular basement membrane thickening, reducing ECM deposition, and ameliorating kidney tissue damage in DN mice (Jiaxin, 2021).

### 2.3 Modulating gut microbiota

The concept of the gut-kidney axis introduces a new paradigm in understanding the interplay between gut microbiota and various kidney diseases. It underscores the significant influence of gut microbiota and their metabolites on the pathophysiology of DN through this axis (Zhao et al., 2023). This involves bidirectional communication between the intestines and kidneys, wherein pathological and physiological changes in either organ can mutually influence and precipitate lesions in the other (Lobel et al., 2020).

A study revealed a markedly lower abundance and diversity of intestinal flora in DN patients compared to healthy individuals. Patients with DN displayed increased levels of *Actinobacteria*, *Bacilli*, *Coriobacteriia*, and *Negativicutes*, whereas *Alphaproteobacteria* and *Clostridia* were found in reduced quantities (Du et al., 2021). Tao et al. (Tao et al., 2019) identified *Escherichia-Shigella* and *Prevotella* as potential microbial markers for distinguishing DN from DM. In *db/db* mice, an increase in the *Firmicutes/Bacteroides* ratio was observed (Singh et al., 2020), alongside decreased levels of *Akkermansia* and *Blautia* (Cani and de Vos, 2017; Liu et al., 2021). These changes might originate from renal dysfunction in DN, which hampers the elimination of purine metabolism by-products such as urea, uric acid, and oxalate, leading to the accumulation of uremic toxins and disruption of the intestinal flora balance (Vaziri et al., 2013). Moreover, DN increases the intestinal pH through ammonia production, causing dysbiosis and an imbalance in intestinal homeostasis, which further affects mucosal stimulation and alters the structure of the intestinal barrier (Richardson et al., 2013; Ramezani and Raj, 2014). Importantly, the intestinal flora and its metabolites play a significant role in the progression of DN (Zhu et al., 2023). Lipopolysaccharide (LPS), an essential component of the outer membrane of Gram-negative bacteria, triggers an inflammatory response and is pivotal in DN development (Salguero et al., 2019). In DN patients with intestinal flora disorder, an overgrowth of Gram-negative bacteria and elevated LPS levels occur, leading to inflammatory responses through the activation of Toll-like receptors 2 (TLR2) and 4 (TLR4) and the nuclear factor kappa B (NF-κB) signaling pathway. This results in the production of inflammatory cytokines like tumor necrosis factor alpha (TNF-α), transforming growth factor beta (TGF-β), and interleukins (IL)-1 and IL-6, contributing to glomerulosclerosis and tubulointerstitial fibrosis, thus exacerbating kidney damage (Griffin et al., 2015; Wu et al., 2019; Mercer et al., 2020). Consequently, treatments targeting gut microbiota modulation have shown potential in restoring kidney function in chronic kidney disease (CKD) (Mao et al., 2023), highlighting therapeutic strategies that involve increasing beneficial bacterial phyla such as *Bacteroidetes* (Albuquerque et al., 2019) and *Akkermansia* (Luo et al., 2022) to counter DN progression.

**FIGURE 3:**
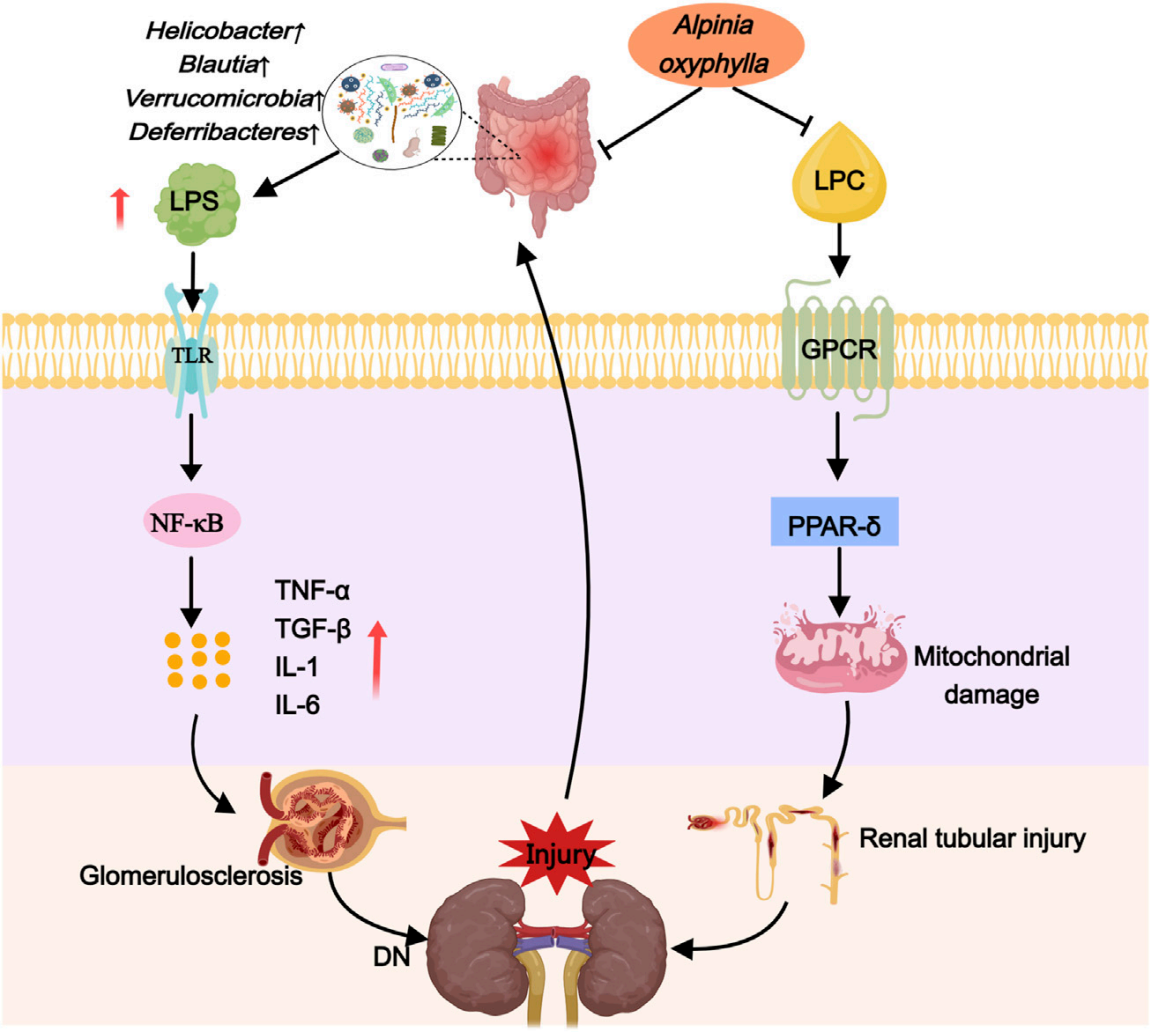
*Alpinia oxyphylla* treats DN by downregulating harmful flora and inhibiting lipotoxicity.

Recent research has demonstrated that *A. oxyphylla* exhibits a promising therapeutic effect on kidney diseases. Compound preparations, individual drugs, and extracts of this plant have been shown to lower blood sugar levels and ameliorate DN by correcting dysbiosis of the gut microbiota. In DN mice, Suoquan Yishen Fang, which primarily features *A. oxyphylla*, modulated the *Firmicutes*-to-*Bacteroides* ratio, enhance the presence of *Cyanobacteria* and *Akkermansia*, and subsequently reduce blood glucose while improving renal function (Ni. 2019). Comparable benefits were observed in DN rats treated with raw or salt-processed forms of *A. oxyphylla*; these preparations decreased the species abundance of *Proteobacteria* and *Enterobacter* while fostering an increase in *Prevotella* and *Rombutella*, leading to improvements in blood glucose, body weight, and renal function parameters (Wu, 2021). Moreover, in T2DM mice, *A. oxyphylla* succeeded in diminishing blood glucose concentrations and significantly mitigating renal pathological alterations by promoting *Bacteroides*, inhibiting *Firmicella* and *Helicobacter* pylori, and boosting the presence of *Akkermansia* (Xie et al., 2018). Consequently, it is suggested that *A. oxyphylla* is instrumental in DN protection through the modulation of gut microbiome composition, the enhancement of beneficial bacterial populations, the suppression of detrimental bacterial communities, and involvement in blood glucose regulation (Figure 3).

### 2.4 Ameliorating lipotoxicity

Lipotoxicity is characterized by the abnormal accumulation of lipids within non-adipose tissues, notably in organs like the kidney that are rich in mitochondria and, consequently, have high energy demands. Lipids, being a primary energy source, tend to accumulate in the kidney. This accumulation particularly affects podocytes and tubular cells within the renal tissue (Mitrofanova et al., 2023). Recent evidence suggests that lipids play a role in causing renal damage through various mechanisms, including damage to podocytes, impairment of tubular function, proliferation of mesangial cells, and activation of endothelial cells. These pathophysiological alterations are thought to be driven by inflammation, oxidative stress, mitochondrial dysfunction, and cell death (Opazo-Ríos et al., 2020; Mitrofanova et al., 2023).

In DN mice, disturbances in lipid metabolism were closely linked to the sphingolipid and glycerophospholipid metabolic pathways, with sphingomyelin, phosphatidylcholine, lysophosphatidylcholine, and phosphatidylethanolamine identified as key metabolites (Ni et al., 2023). Notably, sphingomyelin and phosphatidylcholine have been recognized as predictive biomarkers for DN (Tofte et al., 2019). Elevated serum sphingomyelin levels in DN significantly correlate with adverse clinical outcomes, including a reduced estimated glomerular filtration rate (eGFR), progression to end-stage renal disease (ESRD), and the transition from microalbuminuria to macroalbuminuria (Pongrac Barlovic et al., 2020). Increased phosphatidylcholine levels are implicated in the development of insulin resistance (Kumar et al., 2021) and its diacyl form has been linked to renal dysfunction in individuals with prediabetes (Huang et al., 2021). Lysophosphatidylcholine, a derivative of phosphatidylcholine, detrimentally affects various cell types through G protein-coupled receptor signaling, leading to inflammation, apoptosis, and insulin resistance (Liu et al., 2020). It may also trigger lipid droplet accumulation by overactivating peroxisome proliferator-activated receptor δ (PPAR-δ), resulting in increased expression of the lipid droplet-coating protein Perilipin 2, decreased mitochondrial autophagic flux, and subsequent stress and apoptosis in proximal tubular organelles, ultimately causing a fast deterioration in renal function in DN (Yoshioka et al., 2022). Moreover, phosphatidylethanolamine, a lipid metabolite, is implicated in the early stages of diabetic kidney damage, with considerably higher levels observed in the glomeruli and renal tubules of diabetic patients compared to non-diabetic controls (Wiggenhauser et al., 2022).

Recent research indicates that *A. oxyphylla* can regulate lipid metabolism, aiding in the treatment of DN. Studies have shown that Alpinia oxyphylla can reduce levels of sphingomyelin, phosphatidylcholine, lysophosphatidylcholine, and phosphatidylethanolamine in DN mice. Furthermore, it can enhance the excretion of urinary protein, creatinine, and urea nitrogen within a 24-h urine collection period, and reduce glomerular mesangial cell proliferation and mesangial expansion in these mice (Ni et al., 2023). These findings suggest that *A. oxyphylla* may offer a protective effect for DN by impacting molecular mechanisms associated with lipotoxicity (Figure 3).

### 2.5 Combating oxidative stress

Oxidative stress is instrumental in the development and progression of DN. Chronic hyperglycemia triggers oxidative stress by increasing the production of reactive oxygen species (ROS), reducing antioxidant defenses, causing oxidative damage to DNA and proteins, and prompting the release of inflammatory mediators and cytokines. These mechanisms adversely affect the structure and function of glomerular capillaries and tubules, thereby intensifying renal and systemic damage (Jin et al., 2023). Normally, antioxidants such as reduced glutathione (GSH), vitamin C, and vitamin E, along with antioxidant enzymes including superoxide dismutase (SOD), peroxidase (POD), and catalase, maintain ROS levels essential for cellular health. However, in Type 2 Diabetes Mellitus (T2DM), endogenous antioxidant responses are overwhelmed, leading to ROS accumulation. Compared to control mice, the *db-/db* mice exhibit higher levels of malondialdehyde (MDA), lower GSH levels, and diminished activities of POD and SOD (Xie et al., 2017). Reactive oxygen species also affect the protein phosphatase and tensin homolog (PTEN), an inhibitor of insulin signaling (Hao et al., 2023). An increase in PTEN expression, potentially initiating or worsening DN, is associated with chronic oxidative stress, and inhibition of PTEN can reduce ROS-induced insulin resistance (Butler et al., 2002). DN is marked by tubulointerstitial fibrosis (TIF) and epithelial-mesenchymal transition (EMT), processes in which PTEN plays a vital regulatory role (Khokhar et al., 2020). Furthermore, PTEN contributes to DN pathogenesis by facilitating partial EMT through the action of transforming growth factor beta 1 (TGF-β1) (Li et al., 2019).

It is critical to acknowledge that a Phase 3 international study investigating the treatment of stage G4 DN patients with Bardoxolone Methyl was prematurely halted due to an elevated risk of heart failure (de Zeeuw et al., 2013). Bardoxolone Methyl, which activates the transcription factor Nrf2 to regulate antioxidant genes (Liby and Sporn, 2012), experienced a clinical trial failure. However, this outcome does not necessarily disqualify antioxidant therapy as a controversial approach in diabetes nephropathy management. The trial’s failure may result from the drug’s toxic side effects, dosage concerns, or patients’ pre-existing health conditions, such as high levels of B-type natriuretic peptide or prior hospitalizations for heart failure (Chin et al., 2014). Importantly, subsequent research suggests that excluding high-risk patients allows Bardoxolone Methyl to potentially delay the progression to End-Stage Kidney Disease (ESKD) in Chronic Kidney Disease (CKD) patients (Chin et al., 2018), indicating its potential as a novel treatment for CKD, including DN (Kanda and Yamawaki, 2020). Current studies have determined that oxidative stress contributes to the accumulation of extracellular matrix, endothelial dysfunction, podocyte injury, and DN inflammation, thus exacerbating kidney damage and expediting the progression of DN. This underscores its pivotal role in the advancement of DN (Samsu, 2021).

*Alpinia oxyphylla*, rich in compounds like sesquiterpenes, diterpenes, flavonoids, and diarylheptanes, exhibits strong antioxidant properties (Miao et al., 2015). Research indicates that the extract of *A. oxyphylla* has a concentration-dependent antioxidant effect (Mo et al., 2016). In DN mice, *A. oxyphylla* intake mitigates oxidative stress and reduces PTEN protein expression. It also lowers blood glucose levels, as well as urinary albumin, creatinine, and urea nitrogen concentrations (Xie et al., 2017) (Figure 2).

### 2.6 Mitigating inflammatory responses

Mounting epidemiological and preclinical evidence strongly suggests a link between inflammatory response and the onset and progression of DN. Elevated inflammatory markers, including tumor necrosis factor-alpha (TNF-α), interleukin-1 beta (IL-1β), interleukin-6 (IL-6), and NOD-like receptor protein 3 (NLRP3), have been observed in the serum of DN rats (Fu et al., 2017; Ou et al., 2021). Moreover, patients with type 2 diabetes nephropathy exhibit increased levels of interleukin-18 (IL-18) and IL-1β (Buraczynska et al., 2019; Guo et al., 2023). Importantly, Duan et al. identified inflammation as an independent risk factor for DN progression through renal biopsies (Duan et al., 2021). Experimental models have shown that various anti-inflammatory strategies targeting mediators such as cell adhesion molecules, chemokines, cytokines, and intracellular signaling pathways effectively reduce proteinuria and renal pathology (Rayego-Mateos et al., 2020).

IL-18, a cytokine in the Interleukin-1 (IL-1) family, functions as a predictive marker for diabetic nephropathy (DN) and shows a positive correlation with albuminuria and the progression of renal damage (Fujita et al., 2012; Sueud et al., 2019). Elevated IL-18 levels have been observed in the proximal tubular epithelial cells in renal biopsies from diabetic patients, likely due to the activation of MAPK signaling pathways by diabetic tubular epithelial cells (Miyauchi et al., 2009). IL-1β, a significant innate immune molecule predominantly produced by macrophages, induces the production of other proinflammatory mediators by renal cells (Sims and Smith, 2010) and promotes tubulointerstitial fibrosis, further impairing glycolysis and matrix production (Lemos et al., 2018). The NLRP3 inflammasome, an integral multiprotein complex in chronic inflammation, activates in response to cellular damage (Williams et al., 2022) and plays a vital role in the persistent inflammatory response associated with DN (Wen, Ting, and O'Neill, 2012). Research indicates that hyperglycemia increases NLRP3 expression and activates caspase-1, leading to the release of IL-1β and IL-18 (Wang et al., 2017; Ram et al., 2020), positioning the NLRP3 inflammasome–caspase-1–IL-1β/IL-18 pathway as crucial in the initiation and progression of DN. Notably, Li Kai et al. demonstrated that *A. oxyphylla* suppresses the activation of the NLRP3 inflammasome, caspase-1, and proinflammatory factors such as IL-18 and IL-1β, thereby reducing inflammation and enhancing the survival of human kidney-2 cells (HK-2) (Wang et al., 2022) (Figure 2).

## 3 Conclusion and prospects

As modern society progresses, the incidence of DN continues to increase. The complex pathogenesis of DN makes current medical treatments less effective. Notably, *A. oxyphylla* and its extracts offer significant advantages in managing DN. These natural treatments focus on regulating non-coding RNAs, modulating autophagy and gut microbiota, and reducing lipotoxicity, oxidative stress, and inflammation. Together, these mechanisms alleviate DN symptoms and slow the disease’s progression.

The therapeutic potential of *A. oxyphylla* in DN has been preliminarily validated through animal and cellular studies. Future research should focus on clinical trials to confirm the efficacy of *A. oxyphylla* and its extracts in treating DN patients.

Moreover, the complex interactions between autophagy, noncoding RNAs, the gut microbiome, lipotoxicity, oxidative stress, and inflammation may collectively influence key mechanisms underlying DN. Specifically, reducing NLRP3 inflammasome expression can alleviate podocyte damage not only by decreasing lipid accumulation and reducing lipotoxic effects but also by modulating podocyte autophagy. The PI3K/Akt/mTOR signaling pathway plays a crucial role in important processes such as inflammation, oxidative stress, autophagy, and apoptosis in DN. Future investigations may reveal the effects of *A. oxyphylla* and its compounds on the crosstalk between these cellular pathways in DN from various angles. In the subsequent phase, we will use network pharmacology to pinpoint the advantageous targets of *A. oxyphylla* in DN treatment and proceed with experimental confirmation. This strategy is designed to identify the most efficacious mechanism by which *A. oxyphylla* ameliorates DN.

Crucially, the molecular intricacies of *A. oxyphylla* have not been thoroughly examined, especially concerning its monomeric constituents. Future research should focus on identifying these monomeric components to unveil the essential elements responsible for its therapeutic efficacy in treating DN.

## Abbreviations

Akt, protein kinase B; Atg5, autophagy protein5; Becn1, beclin1; BUN, blood urea nitrogen; COI-IV, collagen type IV; CAT, Catalase; DN, diabetic nephropathy; FN, fibronectin; GBM, glomerular basement membrane; GSH, glutathione; GMCs, glomerular mesangial cells; Glu, blood glucose; GM, glomerular mesangial; GS, glomerulosclerosis; GPCR, G protein-coupled receptor; HK-2, human Renal Cortex Proximal Tubule Epithelial Cells; KDM6A, lysine-specific demethylase 6A; KLF10, kruppel-like factor 10; LC3, Microtuble-associated protein light chain 3; LN, Laminin; LncRNA-PVT1, long noncoding RNA -plasmacytoma variant translocation 1; LPC, lysophosphatidylcholine; LPS, lipopolysaccharide; IL-1, interleukins-1; IL-6, interleukins-6; IL-18, interleukin-16; IL-1β, interleukin-1β; IMD, intestinal microflora diversity; INS, insulin; mTOR, mammalian target of rapamycin; MDA, malondialdehyde; MMP-9, Matrixmetalloproteinase-9; NLRP3, the NOD like receptor protein3; NF-κB, nuclear factor kappa B; PC, phosphatidylcholine; PE, phosphatidylethanolamine; POD, peroxidase; P62, sequestosome1; PI3K, phosphatidylinositol-3-kinase; PI3P, phosphatidylinositol-3-phosphate; PTEN, phosphatase and tension protein homolog; PPAR-δ, peroxisome proliferator-activated receptor δ; ROS, reactive oxygen species; SM, sphingomyelin; SOD, superoxide dismutase; Scr, serum creatinine; TC, total cholesterol; TG, triglycerides; TIMP-1, Tissue Inhibitors of Metalloproteinase-1; TGF-β1, Transforming Growth Factor-beta 1; TNF-α, tumor necrosis factor α; TLR, toll-like receptors; UAER, urinary albumin excretion rate; UACR, urine albumin to creatinine ratio; UAE, urinary albumin excretion; UCr, urea creatinine; ULK1, UNC-51 like kinase 1; ULK2, UNC-51 like kinase 2; α-SMA, α-Smooth muscle actin.
